# Phenotyping ciliary dynamics and coordination in response to CFTR-modulators in Cystic Fibrosis respiratory epithelial cells

**DOI:** 10.1038/s41467-019-09798-3

**Published:** 2019-04-16

**Authors:** M. Chioccioli, L. Feriani, J. Kotar, P. E. Bratcher, P. Cicuta

**Affiliations:** 1 Biological and Soft Systems Sector, Cavendish Laboratory, University of Cambridge, Cambridge, CB3 0HE UK; 2 Section of Pulmonary, Critical Care and Sleep Medicine, Department of Internal Medicine, Yale School of Medicine, New Haven, CT 06510 USA; 3 Institute of Clinical Sciences, Imperial College London, London, SW7 2AZ UK; 4 MRC London Institute of Medical Sciences, London, W12 0NN UK; 5 Division of Cell Biology, Department of Pediatrics, National Jewish Health, Denver, CO 80206 USA

## Abstract

Personalized approaches for systematically assessing ciliary beat dynamics and for drug testing would improve the challenging task of diagnosing and treating respiratory disorders. In this pilot study, we show how multiscale differential dynamic microscopy (multi-DDM) can be used to characterize collective ciliary beating in a non-biased automated manner. We use multi-DDM to assess the efficacy of different CFTR-modulating drugs in human airway epithelial cells derived from subjects with cystic fibrosis (ΔF508/ΔF508 and ∆F508/-) based on ciliary beat frequency and coordination. Similar to clinical observations, drug efficacy is variable across donors, even within the same genotype. We show how our assay can quantitatively identify the most efficient drugs for restoring ciliary beating for each individual donor. Multi-DDM provides insight into ciliary beating responses following treatment with drugs, and has application in the broader context of respiratory disease and for drug screening.

## Abstract (web summary)

Personalized approaches to diagnosis and treatment monitoring could improve the management of cystic fibrosis patients. Here the authors show that multiscale differential dynamic microscopy can assess changes in cilia beating dynamics and coordination in patient-derived airway epithelial cells, in response to different CFTR-modulating drugs, in a patient-specific manner.

## Introduction

Respiratory disorders affect millions of people worldwide and can result from both genetic and environmental causes^1,2^. Many respiratory disorders are characterised by abnormal ciliary beating, be this a causal or derivative behaviour. Current approaches to systematically analysing collective cilia beating are unfortunately limited, as is our understanding of the physical properties required to sustain healthy transport of mucus out of the airways, known as mucociliary clearance (MCC)^3^. Most approaches to phenotyping ciliary motion and coordination are time-consuming and difficult to standardise across labs^4^. Videomicroscopy examination of airway biopsies is often used to estimate ciliary beat frequency (CBF) and inspect waveforms on individual cilia, but this is usually performed manually and requires experienced personnel. Semi-automated approaches to measure CBF have been developed recently; however, even these assess only a subset of the sample and thus cannot detect the broad distribution of CBF that can occur within a given biopsy/culture^5–8^. Furthermore, while CBF is a first and readily accessible phenotype, dysfunctional MCC is responsible for disease pathologies and is, therefore, the ultimate phenotype to be measured and restored in therapy.

A key parameter of healthy MCC is how the ciliary beating is coordinated across large (many cells) distances. Despite its importance, the characterisation of cilia coordination in the context of human respiratory disease has not been well explored. Approaches probing both CBF and cilia coordination have been reported; however, these either require manual selection of the area for analysis^4^ or are not suitable for samples grown in 2D air–liquid interphase (ALI) culture, currently the standard method for culturing in vitro clinical human airway epithelial cells (HAECs) samples^5,9,10^. In ALI culture, the ciliated cells typically exist in patches, and coordination across the entire sample is highly variable^6,11^, precluding, for example, assays based around bead-clearance. As such, there is very little data to describe how collective and coordinated ciliary beating arises in healthy human airway cells, and how this goes awry in disease. We recently reported a video analysis algorithm based on differential dynamic microscopy (DDM), which we called multiscale DDM (multi-DDM)^7^. This allows the characterisation of collective ciliary beating in HAECs in a fast and fully-automated manner. The input required to run the DDM or multi-DDM algorithms are typically 10-s-long bright-field optical microscopy videos of live ALI-cultured cells, taken at 40× magnification and moderately high frame rate (~150 frames per second). By considering the frame differences at various time intervals, transformed in Fourier space, the method extracts temporal and spatial coherence of any dynamics in the video. In particular, for videos with oscillating features such as motile cilia, the CBF is obtained without the need to segment or select regions. Using multi-DDM in ref. ^7^, we demonstrated that the spatial scale of coordinated cilia dynamics can be measured.

We focus this study on cystic fibrosis (CF): a life-threatening genetic disorder caused by loss-of-function in the cystic fibrosis transmembrane conductance regulator (CFTR) protein^8^. Defective CFTR activity in airway epithelial cells leads to loss of airway surface liquid and incompletely hydrated mucin, resulting in a thick layer of mucus that obstructs the airways and promotes chronic bacterial infections and inflammatory lung damage. In individuals with CF, cilia beating is greatly compromised to the extent that the mucus cannot be properly cleared, and the range of cilia movement is severely restricted^8,12^. Of the over 300 disease-causing CFTR mutations that have been identified (www.genet.sickkids.on.ca), only the most common mutations expressed by large groups of subjects have been targeted for drug screening due to the high cost and time-consuming nature of clinical trials. However, a recent study demonstrated the utility of a personalised approach to screen different CFTR-modulating drugs using rectal organoids and the forskolin swelling assay^13^. The success of this study suggests that far more patients with CF might be responsive to existing FDA-approved drugs, if an easily accessible screening assay could be made mainstream.

In the present study, we perform multi-DDM analysis and show that primary HAECs obtained from subjects with the ΔF508/ΔF508 CFTR genotype exhibit unique cilia coordination and CBF dynamics compared to cells from healthy subjects. Multi-DDM data directly quantify the loss of cilia coordination with distance, and how this phenotype is affected by mucus properties in both direct perturbations and pharmacological intervention, providing important information about coordination of cilia dynamics in the context of CF. We apply this approach in a pilot study to assess donor-to-donor variability in response to different CFTR-modulating drugs, specifically the combination of VX-770 (ivacaftor/KALYDECO^®^) and VX-809 (lumacaftor, together termed ORKAMBI^®^), as well as C4, C18 and various combinations thereof. Considerable donor-to-donor variability exists, even in cells obtained from donors with identical mutations in CFTR. Our multi-DDM-based analyses identify the specific drug(s) that best restores collective ciliary beating dynamics for a given donor culture in vitro and thus may be a useful screening tool to predict drug efficacy in vivo in a personalised manner. These data represent an in-depth, quantitative assessment of cilia coordination in HAECs derived from subjects with CF and how coordination changes in response to CFTR-modulating drugs. Although this study applies multi-DDM analysis to investigate ciliary beating dynamics in the context of CF, our approach to phenotyping cilia dynamics could be applied to the other respiratory diseases in which ciliary beating is affected.

## Results

### Multi-DDM analysis of healthy HAECs

**Fig. 1:**
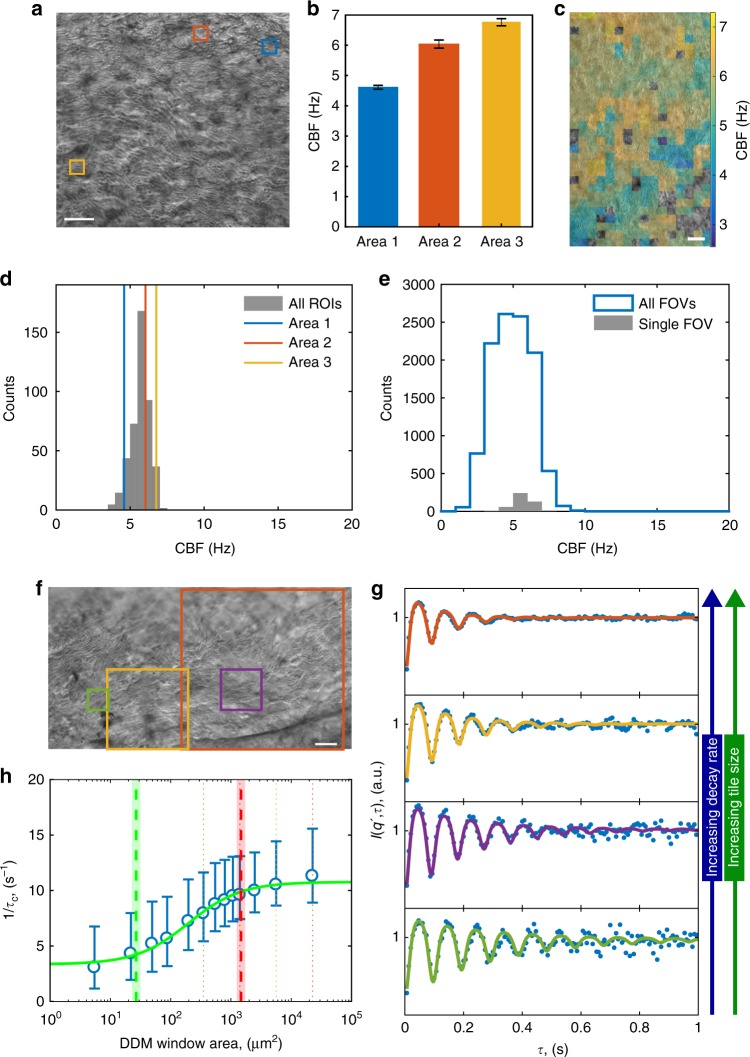
Quantitating ciliary beat dynamics in healthy HAECs via multi-DDM. **a** Example image from a microscopy video showing three different 64 × 64 pixel regions (red, blue and yellow) selected for CBF analysis. Scale bar is 20 μm. **b** Values of CBF in the regions highlighted in **a**. CBFs differed among the regions, highlighting that measuring CBF only in user-selected ROIs is not sufficient for an unbiased and comprehensive measurement. **c** The division in tiles of an FOV, and their measured CBF (in false colours). This yields the full distribution of CBF in this FOV (**d**). CBF measurements from the 3 ROIs of **a**, **b** are shown with vertical lines. **e** A more exhaustive measurement of an ALI sample’s CBF can be obtained by imaging several FOVs scattered around the insert, and aggregating the CBF distributions from all such FOVs (blue outline). In grey, as a comparison, the CBF distribution as in **d**. To measure the coordination length scale of beating cilia, we first divide each FOV into tiles, repeating this process while systematically increasing the size of the tiles. DDM is run on each tile. For example, **f** shows the outline of four tiles, each of a different size. As detailed in ref. ^7^, the output of DDM on motile cilia is an oscillating and decaying signal, examples of which are shown in **g** for each region shown in **f**. We fit the experimental data (dots) with Eq. (2) (solid lines, colours matching the respective regions in **f**) obtaining excellent agreement. The decay rate measures how poorly coordinated the motion within the analysed region is, and it increases with the tile size. Considering the median of the decay rate *τ*_c_^−1^ (circles, whiskers showing 25th and 75th percentiles) measured at each tile area across several FOVs, one obtains a sigmoidal curve that quantitatively represents the length scale of ciliary coordination (**h**). The green and red dashed lines indicate, respectively, the left and right shoulders of the sigmoidal curve (shaded regions indicating the 68% confidence interval). Dotted lines report the area of the tiles highlighted in **f** in matching colour

One of the first parameters normally probed in clinical samples of HAECs, and one which is a general indication of ciliary function, is the CBF. Unlike standard clinical practice whereby a single CBF value is calculated from a user-selected region-of-interest (ROI)^14,15^, multi-DDM provides the user with a distribution of CBF values measured across the imaged fields of view (FOVs). This is important, since CBF can vary across a single sample (Fig. 1a, b), and thus a single point measurement is not an accurate representation of the entire sample. Dividing the entire FOV into square subsets of a pre-defined size (called tiles—64 × 64 pixels, 9.3 μm per side; Fig. 1c) and running the DDM algorithm on each of these tiles generates a distribution of measured CBF across the entire FOV (Fig. 1d, e). In this measurement of CBF, DDM simply extends the concept of probing intensity modulation in a pixel or group of pixels over time, in a systematic and user-free manner.

The standard clinical practice for analysing CBF in ALI cultures does not capture any information regarding the spatial coordination of cilia beating. However, cilia across the airway epithelium must beat in a coordinated manner in order to efficiently propel the mucus layer upwards, and uncoordinated ciliary beating is a hallmark of some ciliopathies, for example Primary Ciliary Diskinesia (PCD)^16–18^. To assess the degree of spatial coordination, we deployed multi-DDM, where the size of the tiles on which the algorithm is run is systematically changed (Fig. 1f): as the tile is decreased in size, cilia beating within the tile becomes more coordinated, which is seen as a decrease in the characteristic decay rate (i.e., the signal decays more slowly) of the resulting signal (Fig. 1g). Plotting the decay rates against their tile size on a semilogarithmic scale produces a sigmoidal dataset that functions as a quantitative measure of cilia coordination within a given sample (Fig. 1h). This data can be fitted to extract a parameter (essentially the position of the sigmoidal curve) that pinpoints the spatial scale of cilia dynamics coordination. The shoulders in the sigmoids are defined as the points where the line that best approximates the central slope meets the respective baselines: the left shoulder (*λ*^2^; Fig. 1h, green dashed line) marks the square of the length scale below which motion is well coordinated, and above which motion starts losing coordination, whereas the right shoulder (*Λ*^2^; Fig. 1h, red dashed line) marks the square of the maximum length within which the sample still shows some coordination. These parameters *λ* and *Λ* are found by fitting the experimental data with the empirical form

1 $$\tau _{\mathrm{c}}^{ - 1} = \frac{a}{{1 + \mu /A}} + c,$$

where *a* and *c* give the level of the plateaus, *A* is the area of the DDM tile, and *μ* is the inflection point of the sigmoid. Since *λ* and *Λ* are separated by a constant shift on the logarithmically spaced *A*-axis, it is possible to use either of the two in this work to compare between different experimental conditions, depending on which one falls within the accessible range of our instrumentation.

### Multi-DDM analysis of CF HAECs

**Fig. 2:**
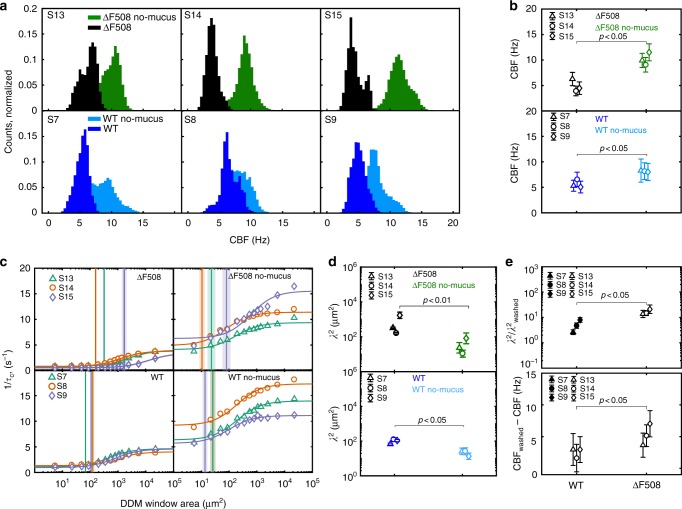
Rheological properties of mucus affect both CBF and length scale of ciliary coordination. **a** Distribution of CBF for both samples from patients with CF (ΔF508/ΔF508) and healthy subjects. Each distribution was built from at least 20 FOVs imaged across the respective sample. **b** Mean and standard deviation for the CBF measured before and after mucus removal on each of the three separate healthy donors (S7, S8, S9) and CF (S13, S14, S15). *p*-value is calculated with paired, one-tailed Student’s *t*-test. **c** Sigmoidal curves of the decay rate *τ*_c_^−1^ as a function of the tile for samples obtained from three subjects with CF (ΔF508/ΔF508) (top) and three healthy subjects (bottom), before and after mucus removal (left and right, respectively). Experimental data are shown as symbols (for the sake of visual clarity, error bars showing 25th and 75th percentiles are omitted), and the sigmoidal fit is shown as a continuous line. The vertical line represents the left shoulder point and marks the coordination length scale squared *λ*^2^, and the shaded area its uncertainty. Each sigmoidal curve is built from up to 20 FOVs imaged across the sample. CF samples are characterised by a higher length scale of ciliary coordination than healthy samples; however, this effect is only apparent in the presence of mucus. **d** Comparison of the *λ*^2^ for each sample shows the decrease in coordination length scale upon washing of the mucus layer. *p*-values calculated with paired, two-tailed Student’s *t*-test. **e** The change in coordination length scale (top) upon mucus washing is significantly different in CF vs healthy samples (unpaired, two-tailed Student’s *t*-test comparing log10 (*λ*^2^)). The same is true for the change in CBF (unpaired, one-tailed Student’s *t*-test). All data shown in this figure were collected on bronchial primary HAECs

In CF, defective CFTR activity leads to loss of airway surface liquid and incompletely hydrated mucin, resulting in a thick mucus layer that greatly restricts cilia beating. We analysed the distribution of CBFs across three different CF patient samples and three different healthy donor samples (bronchial origin) either in the presence or absence of mucus (Fig. 2a, top and bottom panels, respectively). We observed a general trend whereby the shift between the distributions in the presence of mucus and the absence of mucus was greater for CF samples than for healthy samples, although the shift for both was significant (Fig. 2b top and bottom panels). We hypothesised that this was due to the physical properties of the mucus, since the CFTR mutation affects mucus properties and not the intrinsic structure or function of cilia^19,20^.

We next assessed the degree to which mucus affects ciliary coordination in the CF samples (Fig. 2c). The curves generated using the multi-DDM algorithm show the typical sigmoidal shape for both conditions; however, we observed that the position of the left shoulder of the sigmoid (*λ*^2^) differed depending on the presence or absence of mucus (Fig. 2c). This can be seen more clearly in Fig. 2d, which shows the average *λ*^2^ in the presence and absence of mucus for each CF patient. In the absence of mucus, the ciliary coordination length scale for cells of each CF patient decreases to the range typically observed for healthy samples (Fig. 2c–e). The difference in *λ*^2^ observed during the presence of mucus compared to during the absence of mucus is greater for CF samples than for healthy samples (Fig. 2e, top and bottom panels), consistent with the results for CBF. The shift to smaller values represents a decrease in the length scale of ciliary coordination, meaning decreased coordination in the absence of mucus. This shift implies that the mucus, acting like an elastic gel raft, helps cilia to synchronise their dynamics. The difference between the shoulder values obtained from before and after a given perturbation (in this case, removal of mucus) can be used to quantitatively differentiate between ciliary beating dynamics.

### Multi-DDM shows time progression of beating phenotypes

**Fig. 3:**
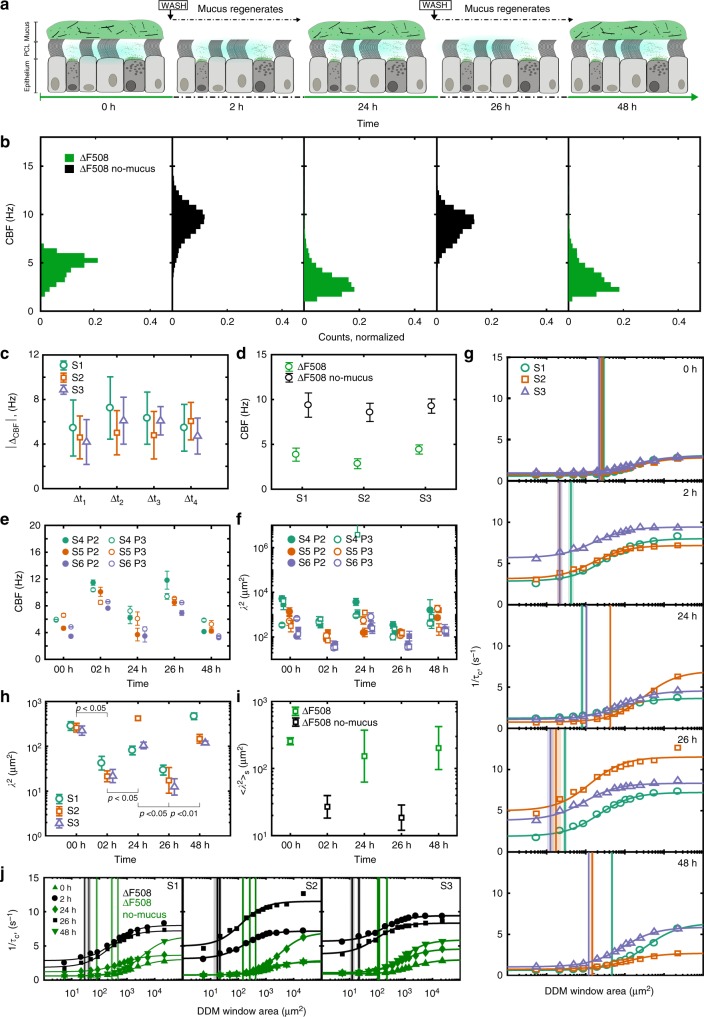
Mucus removal increases CBF and decreases the length scale of ciliary coordination. **a** Experimental timeline of the wash/regeneration experiments on samples obtained from subjects affected by CF (ΔF508/ΔF508). **b** CBF cycled over time between lower CBF in the presence of mucus (green) and higher CBF after the mucus had been removed (black), and returning to lower values once mucus had regenerated (green), repeatedly. Each distribution was built from at least 20 FOVs imaged across samples from subjects S1–S3. **c** The change in CBF upon mucus wash/regrowth was consistent across wash cycles and subject samples (symbols) regardless of the time interval. **d** The weighted average (circles, whiskers mark the uncertainty) of the CBF for each subject sample (S1–S3) across *n* = 3 time points 0 h, 24 h, and 48 h (green, mucus present) and across *n* = 2 time points 2 h and 26 h (black, no mucus) was significantly different between the two conditions, and showed no significant difference between sample subjects. **e** CBF and **f** *λ*^2^ of samples from subjects S4–S6 at different passage numbers (P2 in full markers, P3 empty markers), showing the same cyclical behaviours as S1–S3. In **e**, each marker is the average between the *n* = 2 inserts (whiskers show the maximum error), while in **f** each marker shows *λ*^2^ measured on *n* = 1 insert (confidence interval as error bars). **g** Decay rate (data points in symbols, fit in continuous line) as a function of the DDM tile size. *λ*^2^ (vertical line, its uncertainty as shaded region) consistently decreased after washing the mucus, increasing again after the mucus layer regenerated. This is highlighted in **h**, where *λ*^2^ is plotted against time separately for each subject. *p*-values calculated with unpaired two-tailed Student’s *t*-test, comparing log_10_(*λ*^2^). **i** Geometric mean of *λ*^2^ (squares, geometric SD as whiskers) across *n* = 3 subjects. **j** Plotting all decay rate curves of the same subject (S1–S3) in the same set of axes highlights the shift in *λ*^2^ (vertical lines) dependant on the presence (green) or absence (black) or mucus. All panels show data collected on bronchial HAECs, except for **e**, **f** featuring data from nasal HAECs

The experiments with multi-DDM presented above analysed and compared different HAEC samples maintained under constant cell culture conditions. This is useful to obtain a snapshot comparison between two samples but does not convey information about how the cilia beating phenotype evolves over time in response to various conditions. This is particularly relevant for understanding cellular responses to pathogenic infections and/or pharmacological responses. Having demonstrated the utility of the multi-DDM approach in CBF and cilia coordination analysis in the presence and absence of mucus, we next assessed whether the approach could measure dynamic changes in ciliary beating during successive cycles of washing and mucus regeneration over a 48-h period. Briefly, DDM data were collected from three separate CF donor samples (bronchial origin) in the presence of mucus (0 h) and then immediately after mucus was removed (+2 h), then again once the mucus had regenerated (+24 h). This cycle was repeated at 26 and 48 h (Fig. 3a). We observed complete regeneration of the mucus (and consequently compromised cilia beating) within 24 h, regardless of whether this occurred on the first or second wash. The DDM analysis provides a quantitative measure of this, revealing cyclical changes in CBF over time, with the average CBFs ranging from 2.4 to 5.1 Hz in the presence of mucus, and 8.4–10 Hz once mucus was removed (Fig. 3b, d), consistent with our previous observations. There was no significant difference between the change in CBFs over successive cycles of mucus washing and regeneration or between different donor samples at different passage numbers (Fig. 3e), indicating both the sensitivity (consistent over different time points) and the robustness (consistent across different donors and cell passages) of the DDM approach over time.

The sigmoidal curves generated by DDM analysis are similarly cyclical in nature, and the difference in the coordination length scales is statistically significant and reproducible with successive rounds of mucus removal, across different donor samples, and different passages (Fig. 3f–j). Analysis of the time points immediately prior to and after the removal of mucus produced sigmoidal data that were cyclical in nature and very similar across subjects (Fig. 3g). Consistent with our previous observations, the length scale over which ciliary movement is coordinated decreases upon removal of the mucus and increases once mucus has regenerated. These observations are quantified by plotting the left shoulder values for each subject at the various time points (Fig. 3h, and averaged across subjects in Fig. 3i). As expected, the data from cells in the presence of mucus cluster separately from data following removal of mucus, with a statistically significant difference (*p* < 0.05) in cilia coordination between each two consecutive time points. This analysis shows that the progressive accumulation of mucus increases the length scale over which cilia are coordinated, and that the temporal dynamics of this process can be reliably detected and quantified by multi-DDM. Robustness of the multi-DDM approach is demonstrated by the finding that sigmoidal curves within each subject sample, generated for each condition, cluster closely together (Fig. 3j) and that there is no significant difference in the left shoulder points across subjects in either washed or unwashed conditions (Fig. 3h, j).

### High variability of CBF upon treatment with CFTR-modulators

Having demonstrated the use of multi-DDM to quantify dynamic changes in CBF and coordination with multi-DDM, we reasoned that it might be useful as a way to assess the efficacy of different CF drugs using patient-derived airway cells. A marked improvement in ciliary beating may predict which drug works best for an individual patient, relative to other CF drugs and control treatments. This personalised approach is an important aspect of the assay, as patient-to-patient variation is an obstacle to therapeutic intervention and cannot currently be explained by mutation/s in the CFTR gene alone^21^.

To determine whether the multi-DDM approach could be used to assess drug efficacy in CF, we focused our analyses on CFTR-modulating drugs: small molecules that target the specific defect caused by mutations in the CFTR protein^22^. The ΔF508 mutation affects both protein folding and channel gating, and thus we chose to test the CFTR corrector molecule VX-809 (lumacaftor), the CFTR potentiator molecule VX-770 (ivacaftor) and the combination of both VX-809 and VX-770 (referred to as ORKAMBI), which has been approved by the FDA for use in patients with the ΔF508/ΔF508 genotype. We also tested a set of CFTR correctors from the Cystic Fibrosis Foundation CFTR Chemical Compound Program (cff.org) library, namely C4, C18 and the combination of C4/C18^22–24^.

**Fig. 4:**
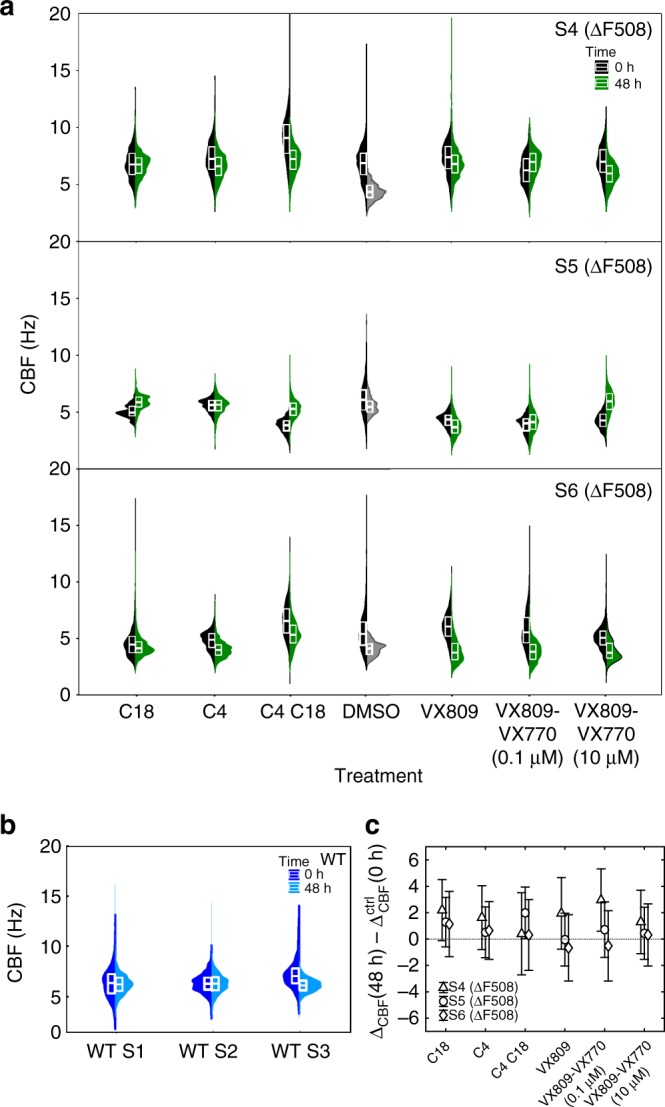
CFTR-modulating drugs yield a relative increase in CBF. **a** Split violin plots showing CBF distribution before (black) and after (green) a 48-h period in which CFTR-modulating drugs were administered to samples from subjects affected by CF (ΔF508/ΔF508). Each distribution pools CBF data from up to 20 FOVs across the sample. **b** Split violin plots showing CBF distribution measured on samples from three different healthy donors. **c** Change in CBF after 48 h treatment compared to the change in CBF of the DMSO control over the same amount of time. The CFTR-modulating compounds increase the CBF compared to the DMSO control in all but three cases. Markers show the average value, error bars are obtained by propagating the SD of the individual CBF distributions. Each marker, with its error bars, pertains to a single subject. All data are from nasal HAECs

A single treatment of C4 (10 µM), C18 (5 µM), C4/C18 (10 µM/5 µM), VX-809 (3 µM), VX-809/VX-770 (3 µM/0.1 µM/) and VX-809/VX-770 (3 µM/10 µM) (hereafter referred to as the panel of drugs) and dimethyl sulphoxide (DMSO)-only control was administered to HAECs (nasal origin) from three CF subjects (F) and data were collected at 0 and 48 h (Fig. 4a). As expected, DMSO-only control treatments for all three CF patient samples resulted in a decreased CBF range at 48 h compared to 0 h (Fig. 4a). We attributed this to the accumulation of mucus in these samples over the 48 h period. In contrast, the same CF patient samples that were treated with CFTR-modulating drugs generally did not show a decrease in the CBF range over the 48 h period, although some exceptions to this were observed (e.g. in patient S6 treated with C4 or VX-809, and in patient S4 treated with C4/C18). In all but three cases, however, CFTR-modulating compounds yielded either an increase in CBF or a less marked decrease when compared to the DMSO-only control (Fig. 4c). In general, even though the mean CBF at 0 and 48 h often differed (see internal box plots Fig. 4a), the range of CBFs at a given time point was much larger than the mean and frequently overlapped between time points, even on a single sample. We also collected data from three untreated, healthy subject controls (Fig. 4b). Again, the range of CBFs within a given sample was considerable, although in general CBF for untreated healthy controls was higher than for CF subject samples, consistent with previous reports^5,10,25^. The range of CBFs observed both within a given sample and between samples of the same genotype supports a growing consensus in the field that CBF is variable and may not be a reliable read-out of ciliary beating dynamics, especially in the context of disease^26^.

### Multi-DDM tracks the beating response to CFTR-modulators

In our previous results, we showed that removal of mucus from CF patient samples resulted in a decrease in the length scale of coordination, meaning that without mucus, cilia were less coordinated at a given scale than when mucus was present. We interpret this as the loss of highly coupled movement imposed by the thicker mucus in CF, which in turn allows cilia to beat more freely. Cells grown in 2D ALI culture do not beat uniformly. Thus, a decrease in the length scale of coordination is due to the fact that cilia are able to beat more freely but, when analysed across the entire FOV, do not do so synchronously. The same rationale applies to drug screening. If the drug is effective, cilia will be less restricted in their movement and able to beat more freely. When analysed across the entire FOV, this will manifest as a decrease in the length scale of coordination, since the freely-beating do not do so uniformly.

**Fig. 5:**
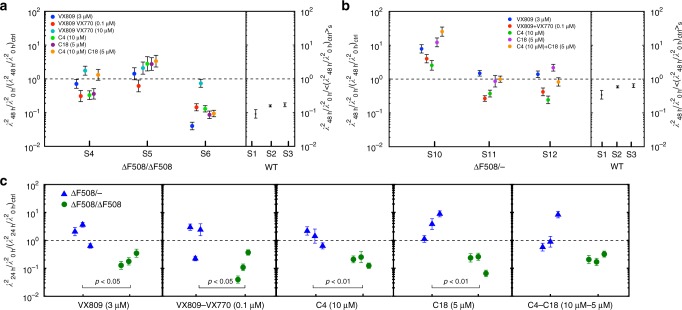
Ciliary beating coordination length scale upon CFTR-modulating drugs is patient dependent. The response to 48 h treatment with CFTR-modulating drugs is shown in **a** and **b** for samples from subjects of ΔF508/ΔF508 and ∆F508/− genotype, respectively. Samples from subjects with ΔF508/ΔF508 genotype responded better as a group to the same panel of drugs after 24 h of treatment. This is shown in **c** where we plot the response to the same CFTR-modulating compound for subjects of both genotypes. Samples from ΔF508/ΔF508 subjects had their coordination length scale moved significantly further towards healthy phenotype values than samples from ∆F508/− subjects. *p*-value was calculated with unpaired, one-tailed Student’s *t*-test on log_10_(*λ*^2^). Significance was not found only in the difference in response to the combination of C4 and C18. Error bars show the uncertainty on the response measurements. Each data point in CF data pertains to one compound-treated sample, normalised by the control sample from the same subject. Healthy data were instead normalised by the average change in coordination length scale in samples of the relevant CF genotype. All data are from nasal HAECs

To test this, we treated HAECs (nasal origin) derived from CF patients (ΔF508/ΔF508 and ∆F508/−) with a panel of CFTR-modulating drugs. Analysis of the coordination of ciliary beating in CF cells treated with the panel of CFTR-modulating drugs shows that, on average, the compounds tested had a positive effect on the samples, reducing their coordination length scale or slowing down its increase when compared to the DMSO-only control (Supplementary Figure 1). However, when analysing the response amongst the cultures from different donors, one can see how the response varies (Fig. 5a, b and Supplementary Figure 2). No one drug produced a consistent response (i.e., consistent shift in coordination length scale, compared to the shift in the control) across the three patients of either genotype. In contrast, each patient did appear to be relatively consistent with regard to whether or not they were generally responsive to the panel. For example, CF ∆F508/− patient sample S10 showed increased length scale of coordination for all drugs when compared to the control (data points above the reference line), and ΔF508/ΔF508 patient sample S5 for all but one drug, suggesting that these patients may not be particularly responsive to CFTR-modulators. In contrast, CF ΔF508/ΔF508 patient sample S6 showed a decreased length scale of coordination for all drugs in the panel relative to DMSO, suggesting that CFTR-modulators may be an effective treatment for this patient. In this case, VX-809 alone showed the greatest decrease in coordination length scale relative to DMSO, whilst a high (10 μM) concentration of VX-809/VX-770 was least effective, roughly on par with DMSO, consistent with previous reports^27,28^. CF ΔF508/ΔF508 patient sample S4 and CF ∆F508/− patient samples S11 and S12 showed mixed responses, with VX-809/VX-770 and C4 the most consistently effective.

Given that the CF ∆F508/− genotype encodes roughly half the amount of defective CFTR protein compared to the CF ΔF508 genotype, we reasoned that, in general, CFTR-modulating drugs would not be as effective for the ∆F508/− genotype as for the ΔF508 genotype. We observed a significant difference between the response of the three CF ΔF508/ΔF508 samples as a group, compared to the three ∆F508/− samples as a group for all drug treatments relative to DMSO (Fig. 5c). The response is measured as the change in *λ*^2^ in the treated sample (*λ*^2^_48 h_/*λ*^2^_0 h_) normalised by the change in *λ*^2^ measured in the DMSO control sample from the same subject, (*λ*^2^_48 h_/*λ*^2^_0 h_)_ctrl_. Points below the reference *λ*^2^ = 1 line show samples where the treatment succeeded in moving the coordination length scale towards values characteristic of a healthy phenotype and further from the DMSO control. Points around or above the reference line show samples on which the treatment had no positive effect on the samples. The data are compared to the change in coordination length scale on healthy samples normalised by the average change in coordination length scale in samples of the relevant CF genotype. Samples from ΔF508/ΔF508 subjects had their coordination length scale moved significantly further towards healthy phenotype values than samples from ∆F508− subjects.

## Discussion

Probing collective cilia beating dynamics across cells at ALI with multi-DDM provides direct quantitative phenotyping of cilia function and represents a useful approach to determine the coordination of cilia and their efficiency in propelling mucus. Our framework is not limited to the estimation of the CBF in ALI culture, which we found to be highly variable, but also gives information about the coordination and the spatio-temporal correlation of the ciliary beating as well. This is demonstrated by the analysis of cilia coordination either in the presence or absence of mucus. In cells derived from subjects with CF, the very thick and stiff mucus increases the length scale over which ciliary movement is correlated.

In this study, we encountered two instances where our analysis was limited by extremely thick mucus: firstly, at the perimeter of the well insert in ALI culture, where mucus accumulates more rapidly than on the rest of the insert membrane. This was easily overcome by avoiding this area for data collection. The second instance in which thick mucus was limiting was in the context of CF, where cells accumulated excessive amounts of mucus when left without intervention for several days. We observed this in our vehicle-only controls during the drug treatment experiments. After 48 h, motion in the FOV for vehicle-only HAECs showed a very high length scale of coordinated ciliary beating, which made it impossible to use the right shoulder of the sigmoidal curve (which marks the length scale at which all coordination is lost) as it fell out of the detection range. Instead, we used the left shoulder, which represents the length scale above which perfect coordination begins to be lost. Focusing on relative measurements that compare the positions of either the left or right shoulder point, and not the absolute values, affords greater flexibility across a range of different conditions as either the left or right shoulder point can be chosen, depending on the specific circumstances and equipment. An additional consideration is the proportion of ciliated cells to secretory cells. Not only does this affect the production of mucus but also affects the number of beating cilia that can be analysed. This effect is mitigated, at least in part, by the fact that the multi-DDM approach is based on the analysis of a single culture of differentiated cells before and after treatment, and thus the proportions should remain constant throughout the analysis.

The results of our study suggest that dynamic ciliary coordination may be a better way to assay drug efficacy than CBF. CBF is known to be highly variable, sensitive to parameters such as cell type, tissue of origin, temperature and adherence^29,30^. Indeed, we found that the range of CBFs was high within a single sample derived from both healthy controls and CF patients. Although in some cases the mean CBF was different between conditions, our analysis of the distribution across the entire sample showed substantial overlap. These data add to accumulating evidence that CBF alone is not a reliable read-out of ciliary beating dynamics^26,31^. We found that ciliary coordination was a sensitive, robust indicator of ciliary beating dynamics over time in the presence or absence of mucus.

A key issue that became evident from our study was the extent to which individual patients with the same CF genotype exhibit variation in their response to CFTR-modulating drugs. Although CF is monogenic, caused by a mutation in CFTR, it is well established that this does not account for the full spectrum of phenotypic variability observed in CF patients, including responsiveness to drugs^32–34^. In our study, at least one patient for each genotype proved to be refractory to essentially all CFTR-modulating therapies tested, despite the fact that our panel included VX-809/VX-770, which are the components of ORKAMBI, the FDA-approved treatment option for CF patients with the ΔF508/ΔF508 genotype. Expanding the panel of drugs to include more CFTR-modulating drugs as well as mucolytics and other therapeutic agents could potentially provide alternative therapeutic options for such patients. Understanding why certain subjects respond differently to various drugs despite sharing a common CFTR mutation may also help to uncover genetic factors that contribute to phenotypic variability in CF. In the future, it will be necessary to incorporate clinical data to build correlations between the drug response as captured by multi-DDM and the bonafide in vivo response of the patient.

We envision a number of applications for the multi-DDM approach presented here. Firstly, it should now be possible to quantitatively assess a wide range of putative drugs for the treatment of CF in a personalised manner, based on changes in ciliary beating and coordination of the patient’s own cells. In vivo, MCC is mediated by the coordinated action of thousands of beating cilia, and so a quantitative approach to measure ciliary beating dynamics is highly suited to the analysis of therapeutic modulators of MCC. This is particularly relevant for drugs such as ENaC inhibitors, mucus hydrators and DNases/mucus disruptors, which do not modulate CFTR function, and so cannot be tested using Ussing chamber analysis nor the Forskolin-induced swelling assay^35^. A second application of our approach is in the analysis of CFTR-modulating drugs, where it may be useful as a complementary assay alongside Ussing chamber analysis. Theoretically, restoration of CFTR ion channel function as measured by Ussing chamber analysis should lead to mucus rehydration and increased MCC. In practice, however, the degree to which CFTR activity is restored varies among patients, and the relationship between the Ussing chamber results and the observed clinical response is not always clear. In the future, it will be important to investigate whether there is any relationship between the data obtained via Ussing chamber, multi-DDM and in vivo efficacy to see if there is a threshold of ion transport that is required in order to improve MCC.

In conclusion, we have developed a straightforward and quantitative assay based on multi-DDM to characterise and detect changes in ciliary beating of HAECs grown in ALI culture. Our approach improves on previous methods of ciliary beat analysis in that it is unbiased and automated, and because it captures the spatio-temporal coordination of collective cilia beating which is crucial for MCC, and not simply the CBF. Our pilot study applies multi-DDM to HAECs obtained from subjects with the ΔF508/ΔF508 or ∆F508/- mutations in CFTR to assess their responsiveness to a panel of CFTR-modulating drugs. Our data show that patient-to-patient variability in drug response is considerable, even for patients that share common mutations in CFTR. The multi-DDM approach allows for the fast and efficient identification of compounds that result in restored ciliary beating dynamics in a completely personalised way. Finally, since multi-DDM is ultimately a means to assess cilia beating dynamics, its use is not limited to CF but may be applied to other diseases in which ciliary beating is affected.

## Methods

### Study design

The overall objective of this study was to use the recently developed multi-DDM algorithm to characterise ciliary beating in cells from subjects with CF, and to test whether the approach was sensitive enough to detect changes in ciliary beating over time and in response to various drug treatments. The complete dataset used in this work is presented in Supplementary Table 1. Initially, we applied multi-DDM to primary bronchial HAECs and manipulated the mucus layer to affect ciliary beating. HAECs feature in Figs. 1, 2 and 3 (except for panels e and f). Once our approach was validated—i.e., it became clear that the data obtained from multi-DDM analysis could be used to distinguish between different samples under different conditions—we obtained primary clinical samples for experiments involving CFTR-modulating drug treatments. For every experiment, the DDM analysis was based on data collected from HAECs obtained from three different subjects, either healthy subject controls, or subjects harbouring the ΔF508/ΔF508 mutations in CFTR, or heterozygous subjects with ∆F508/− mutations. We chose to focus on the ΔF508 mutation as it is the most common cause of CF and one for which at least one therapeutic drug has been approved (ORKAMBI^®^). At least 20 videos were taken of each of the samples at each data collection point, avoiding the perimeter of the cell culture insert due to the accumulation of mucus in that region (an experimental artefact caused by the geometry of the vessel). Multi-DDM analyses were performed as previously described^7^ and in a such a manner as to ensure the entire FOV was represented in an unbiased manner. This study was not blinded.

### Human material and cell culture

HAECs obtained through the clinic were isolated from nasal brushings of the inferior turbinate performed using a cytology brush on CF subjects attending the Adult Clinic at National Jewish Health (Denver, CO, USA). Samples were collected in compliance with all relevant ethical regulations using a protocol approved by the Institutional Review Board at National Jewish Health, and all subjects provided written informed consent. The median age of the donors was 29 years, 5/9 were female, and all were Caucasian and either healthy, or of the CFTR genotype ΔF508/ΔF508 or ∆F508/−. Nasal epithelial cells obtained from brushings were expanded in culture using previously described methods of conditional reprogramming culture (CRC)^36^. Briefly, nasal brushing samples were dissociated in Dulbecco’s phosphate buffered saline solution containing 5 mM EDTA and 5 mM EGTA and mechanically processed by repeatedly pipetting. After washing, cells were resuspended in CRC medium containing the rho-kinase inhibitor, Y-27632, and plated on a feeder cell layer of gamma-irradiated 3T3 fibroblasts. Reprogrammed epithelial cells were expanded at 37 °C in a humidified incubator in an atmosphere containing 5% CO_2_, removed using two-stage trypsinisation, and re-plated on a fresh feeder layer. After reaching ~80% confluence, passage 2 cells were aliquoted and stored in liquid nitrogen. Later, thawed cells were plated onto feeder layers in CRC media with Y-27632 and expanded until ~80% confluence was reached.

After a two-stage trypsinisation and treatment with DNase I, cells were plated onto collagen-coated permeable supports in a 24-well format (Corning Incorporated, Tewksbury, MA, USA) at a density of 100,000 cells/cm^2^. Once confluence was reached, apical media was removed and basal media was switched to PneumaCult ALI media (STEMCELL Technologies). Cells formed well-differentiated cultures after 4–6 weeks at the air–liquid interface. HAECs obtained from Epithelix (MucilAir™, Epithelix Sàrl, Geneva, Switzerland) were isolated from bronchi and cultured as per the manufacturer’s instructions. General cell culture maintenance involved washing the cells twice a week as previously described^6,37^. Briefly, the apical surfaces of the cultures were incubated for 20 min in 200 µl of sterile phosphate-buffered saline (PBS; GIBCO^™^). After 20 min, the solution was gently removed by suction pipette. For the mucus removal experiments (Fig.2), the apical surfaces of the cultures were washed twice in PBS. For the CF cells, the wash also contained 1 mM Dithiothreitol (DTT) solution (Sigma Aldrich, St. Louis, MO) and was followed by two additional PBS washes to remove all free DTT and remaining mucus.

### CFTR-modulating drug assays

Solutions of VX-809, (lumacaftor; Selleck Chemicals LLC, Huston, USA), VX-809–VX-770 (ivacaftor; Selleck Chemicals LLC, Huston, USA), C4, C18 and C4–C18 were prepared in DMSO (Sigma) at the following concentrations: VX809 3 mM, VX770 0.1 mM and 10 mM, C4 10 mM. C18 5 mM. Each solution was then diluted 1000-fold in culture media and added basolaterally to HAECs in ALI culture at 0, 24 and 48 h. The control treatment consisted of DMSO 0.1% (Sigma).

### Video acquisition

At least 20 videos were acquired from each sample at each time point on a Nikon Eclipse Ti-E inverted microscope (Nikon Instruments, Japan) with a 40× objective (Plan Apo λ 40×, N.A. 0.95, Nikon). Digital high-speed videos were recorded under bright-field illumination at a sampling frequency of 150 fps using a Grasshopper^®^3 GS3-U3-23S6M-C CMOS camera (FLIR Integrated Imaging Solutions GmbH, Germany). Samples of epithelial cells were imaged in a custom-made chamber, where temperature, CO_2_ and humidity were continuously monitored and maintained at values of 37 °C, 5 and 90% respectively. Any videos that showed drifting or duplicated areas of analysis were not included in the analyses (leaving on average 14 ± 7 videos).

### Data processing

Videos were uploaded to our custom multi-DDM algorithm pipeline (coded in MATLAB, the MathWORKS) and processed as described in greater detail in ref. ^7^. Briefly, the DDM algorithm prescribes to take the algebraic difference of several couples of frames separated by a lag time *τ*. These differences are then Fourier transformed in space, and the results averaged, to yield an averaged 2D power spectrum. If the anisotropy of the sample’s dynamics is not of interest for the analysis, an azimuthal average of the 2D power spectrum is taken. By repeating the process for different values of the time lag *τ*, we build the Image Structure Function *I*(*q*,*τ*)^38,39^. As detailed in ref. ^7^, we then extract information about the dynamics of the sample by fitting the *I*(*q*,*τ*) with an empirical function:

2 $$I\left( {q,\tau } \right) = A\left[ {1 - {\mathrm{exp}}({\mathrm{cos}}(2\pi \nu \tau ))} \right]\exp ( - \tau /\tau _c) + B.$$

The frequency of oscillations *ν* is the CBF, and the decay rate *τ*_c_^−1^ measures the degree of coordination within the FOV: the lower the decay rate, the higher the coordination of the movement. The MATLAB code we developed performs the DDM algorithm automatically on the biggest square region that the FOVs can contain, and on each tile of increasingly finer square grids. The software produces an output file per video. Each output file contains the result of the DDM algorithm for each of the tiles (of all sizes) analysed. The software also gives the user a list of the tiles that were covering a region of the sample with little or no motion, and/or where the fitting of *I*(*q*,*τ*) failed or was deemed unreliable. These tiles were not used for subsequent analysis.

The motion detection used for the experiments in Figs. 1–3 is described in ref. ^7^ and relies on thresholding an image obtained by taking the standard deviation of the fluctuation of each pixel’s recorded grey level over time. This simple approach works well in most cases, but struggled in some videos during the experiments featured in Figs. 4, 5, because it did not discard some DDM tiles where there was no ciliary motion. We therefore devised a different motion detection algorithm, geared towards better performances in case of low signal-to-noise ratio. In this revised algorithm, we take differences between every other frame, to highlight regions where the sample shows fast motion. These images undergo a median filtering, to reduce the effect of camera noise, and a local standard deviation filtering. This last filter highlights regions with high alternation of dark and bright pixels, where the moving cilia are located. We then take the base-10 logarithm of the standard deviation across all these filtered images. The motion map thus obtained is bright in correspondence of moving cilia, and dark over static regions of the sample. Segmentation then finds the foreground features, where the beating cilia are. This is done by fitting the dark end of the pixel values histogram with a Gaussian, and marking as background (so, no moving cilia) all pixels with value smaller or equal than the sum of mean and width of the fitted Gaussian. This algorithm was seen to reliably work as long as some static regions are present in the FOV. Once all videos taken on a sample were automatically analysed, we collected the CBF values measured on DDM tiles (of size closely matching a cell size, so 64 pixels, or 9.3 μm side) across all videos pertaining to the same sample to build the CBF distributions.

The sigmoidal curves showing the loss of coordination upon increasing the DDM tile size are built by pooling together data from many FOVs on the same sample. For each analysed tile size, we build the distribution of values of the decay rate *τ*_c_^−1^ measured on tiles of matching size across all videos on the same sample. The marker in the plots shows the medians of such distributions, while the whiskers show the 25th and 75th percentiles (see an example in Fig. 1h and Supplementary Figure 2). The experimental data points are then fitted as

$$\tau _{\mathrm{c}}^{ - 1} = \frac{a}{{1 + \mu /A}} + c,$$

where *a* and *c* give the level of the left and right plateaus, *A* is the area of the DDM tile, and *μ* is the inflection point of the sigmoid. The left and right shoulders of the sigmoidal curve are then the abscissas $\left( {\lambda ^2 = \mu {\mathrm{e}}^{ - 2}\,{\mathrm{and}}\,\varLambda ^2 = \mu {\mathrm{e}}^2} \right)$ of the points where the line that best fits the central slope meets the prolongation of, respectively, the left and right plateaus.

### Statistical analyses

When not showing the full distribution, CBF results on a single sample are presented as mean ± standard deviation (SD) of the CBF values measured across all the tiles that showed movement across all the imaged FOVs (Fig. 2b). When averaging across different subjects or inserts, the figure captions specify how the error bars were obtained. Changes in CBF are measured as $\Delta {\mathrm{CBF}} = {\mathrm{CBF}}(t) - {\mathrm{CBF}}(t = 0)$ and displayed as marker and error bars showing, respectively, $\mu _{\mathrm{\Delta }} = \mu _t - \mu _0$ and $\sigma _{\mathrm{\Delta }} = \sqrt {\sigma _t^2 + \sigma _0^2}$, with *μ* and *σ* being the mean and SD of the relative distributions (Fig. 2e—bottom, 3c). The same process was employed in Fig. 4c, where we plot ${\mathrm{\Delta CBF}} - {\mathrm{\Delta CBF}}_{{\mathrm{ctrl}}}$.

*λ*^2^ measurements on a single sample are presented as measurement ±68% confidence interval (Figs. 2d, 3f). The confidence interval was treated as an uncertainty and propagated as such when combining measurements taken at different times or on different samples (Fig. 2e—top, 5). Averages across samples or subjects are presented as geometric mean ± geometric SD (Fig. 3h–i), unless otherwise specified in the figure caption. Statistical analysis was performed by paired or unpaired two-tailed Student’s *t*-test using MATLAB. The 95% confidence level was considered significant. Details about *p*-values and the test used to calculate them are given in the figure captions.

## Supplementary information

Supplementary Information

## Data Availability

The data that support the findings of this study are available from the corresponding authors upon reasonable request.

## Code availability

The code used for running multi-DDM analysis is available at 10.17863/CAM.37743.
