# Supplementary material for: Phenotyping ciliary dynamics and coordination in response to CFTR-modulators in Cystic Fibrosis respiratory epithelial cells
Source: Nat Commun. 2019 Apr 16;10:1763. doi: 10.1038/s41467-019-09798-3 (PMC6467870; doi:10.1038/s41467-019-09798-3)
Supplement: Supplementary file 1 — Supplementary Information [file 41467_2019_9798_MOESM1_ESM.pdf]

Supplementary Materials of:

Phenotyping ciliary dynamics and coordination in response to CFTR-modulators in Cystic Fibrosis respiratory epithelial cells

M. Chioccioli[1,2*], L. Feriani[1,3,4*], J. Kotar[1], P. E. Bratcher[5ǂ], P. Cicuta[1ǂ]

[1]Biological and Soft Systems Sector, Cavendish Laboratory, University of Cambridge, Cambridge CB3 0HE, UK.

[2]Section of Pulmonary, Critical Care and Sleep Medicine, Department of Internal Medicine, Yale School of Medicine, New Haven, CT, USA.

[3]Institute of Clinical Sciences, Imperial College London, London, UK.

[4]MRC London Institute of Medical Sciences, London, UK.

[5]Division of Cell Biology, Department of Pediatrics, National Jewish Health, Denver, CO, USA.

ǂ To whom correspondence should be addressed

* These authors contributed equally.

This file contains two supplementary data figures and a table of the samples used in the work:

Supplementary Figure 1: Patients-averaged response to tested compounds.

Supplementary Figure 2: Sigmoids for ΔF508/ΔF508 and ΔF508/- treated with CFTR-modulating drugs (Figure 5)

Supplementary Table 1: Samples used throughout the study

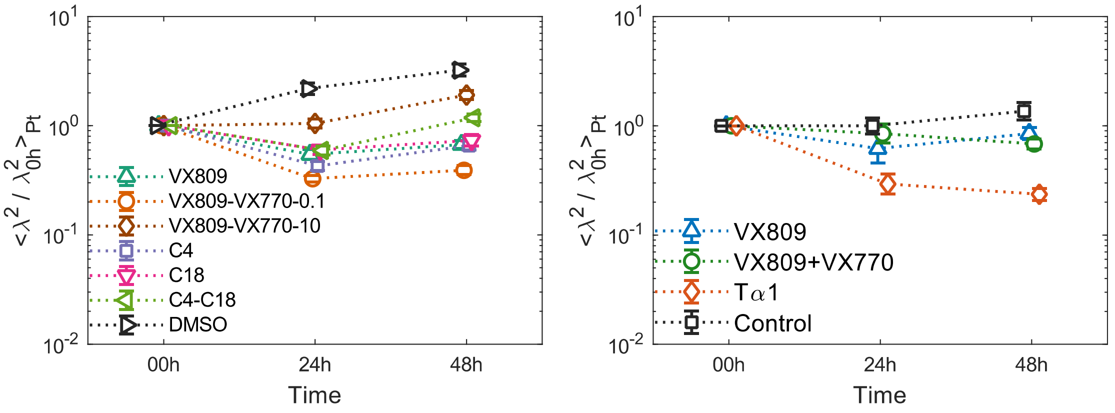

Supplementary Figure 1: ***CFTR-modulating drugs yield on average a reduction in λ^2 when compared to the DMSO only control.*** *(left) Change in coordination length scale during the treatment with CFTR-modulating drugs detailed in Figure 4 and 5 of the main text, averaged across donors. λ^2 either decreases over time or increases, but less than the DMSO-only control. (right) Change in λ^2 in a preliminary experiment done in similar conditions, but where we had washed the inserts at t=-48h. Thymosin α1 (Tα1, CRIBI Biotechnology, Padova, Italy) was reconstructed in MilliQ water at 100μg/ml concentration [1,2], diluted 1000-fold in culture media and added basolaterally to HAECs in ALI culture at 0, 24, and 48. The role of Tα1 as a CFTR modulator is not universally accepted [2–4], and while in our experiments it did seem to have a beneficial effect, multi-DDM does not provide any information about its mode of action.*

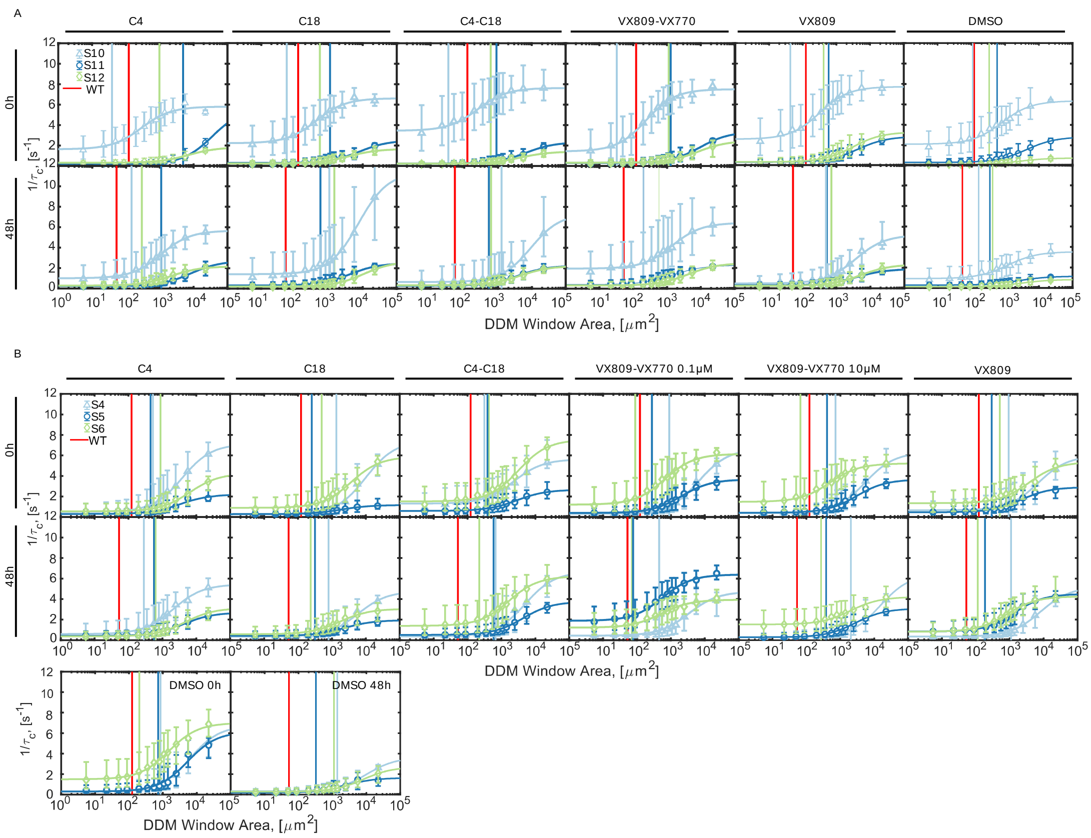

Supplementary Figure 2: "Raw sigmoid" data for (A) ΔF508/- treated with CFTR-modulating drugs and (B) ΔF508/ΔF508 treated with CFTR-modulating drugs (data of Figure 5).

Sample ID	Figure	Genotype	Provenance	Patient	Passage	origin (nasal/bronchial)	Sex	age
S13	2	F508del/F508del	Epithelix			bronchial		
S14	2	F508del/F508del	Epithelix			bronchial		
S15	2	F508del/F508del	Epithelix			bronchial		
S7	2	Healthy	Epithelix			bronchial		
S8	2	Healthy	Epithelix			bronchial		
S9	2	Healthy	Epithelix			bronchial		
S1	3	F508del/F508del	Epithelix			bronchial		
S2	3	F508del/F508del	Epithelix			bronchial		
S3	3	F508del/F508del	Epithelix			bronchial		
S4	3E,F	F508del/F508del	NJH	Pt026	P2	nasal	Female	27
S4	3E,F	F508del/F508del	NJH	Pt026	P3	nasal	Female	27
S5	3E,F	F508del/F508del	NJH	Pt030	P2	nasal	Female	35
S5	3E,F	F508del/F508del	NJH	Pt030	P3	nasal	Female	35
S6	3E,F	F508del/F508del	NJH	Pt051	P2	nasal	Male	29
S6	3E,F	F508del/F508del	NJH	Pt051	P3	nasal	Male	29
WT S1	4,5,S2	Healthy	NJH	Pt129	P2	nasal	Male	28
WT S2	4,5,S2	Heathy	NJH	Pt080	P2	nasal	Male	32
WT S3	4,5,S2	Healthy	NJH	Pt153	P2	nasal	Male	34
S4	4,5,S2	F508del/F508del	NJH	Pt026	P2	nasal	Female	27
S5	4,5,S2	F508del/F508del	NJH	Pt030	P2	nasal	Female	35
S6	4,5,S2	F508del/F508del	NJH	Pt051	P2	nasal	Male	29
S10	5,S2	F508del/null	NJH	Pt082	P2	nasal	Female	27
S11	5,S2	F508del/null	NJH	Pt109	P2	nasal	Female	28
S12	5,S2	F508del/null	NJH	Pt152	P2	nasal	Female	30

Supplementary Table 1: samples used in the study

Bibliography

1. Romani, L. *et al.* Thymosin α1 represents a potential potent single-molecule-based therapy for cystic fibrosis. *Nat. Med.* **23,** 590–600 (2017).

2. Romani, L. *et al.* Reply to 'F508del-CFTR is not corrected by thymosin α1'. *Nat. Med.* **24,** 891–893 (2018).

3. Tomati, V. *et al.* Thymosin a-1 does not correct F508del-CFTR in cystic fibrosis airway epithelia. *JCI Insight* (2018). doi:10.1172/jci.insight.98699

4. Matthes, E., Hanrahan, J. W. & Cantin, A. M. F508del-CFTR is not corrected by thymosin α1. *Nat. Med.* (2018). doi:10.1038/s41591-018-0080-0
